# A mathematical model for predicting the adult height of girls with advanced puberty after spontaneous growth

**DOI:** 10.1186/1471-2431-14-172

**Published:** 2014-07-03

**Authors:** Pierre Lemaire, Delphine Pierre, Jean-Baptiste Bertrand, Raja Brauner

**Affiliations:** 1 Université Grenoble Alpes, G-SCOP, F-38000 Grenoble, France; 2 CNRS, G-SCOP, F-38000 Grenoble, France; 3 Université Paris Descartes and Fondation Ophtalmologique Adolphe de Rothschild, 75940 Paris, France; 4 Service de pédiatrie et néonatologie, Centre hospitalier de Courbevoie-Neuilly-Puteaux, 92205 Neuilly sur Seine, France

## Abstract

**Background:**

Advanced puberty in girls is defined as the onset of puberty between the ages of 8 yr and 10 yr. The objective was to predict adult height (AH) at initial evaluation and to characterize patients with an actual AH below -2 SD (152 cm) and/or lower than their target height (TH) by > one SD (5.6 cm).

**Methods:**

Data analysis using multiple linear regression models was performed in 50 girls with advanced puberty who reached their AH after spontaneous puberty.

**Results:**

The actual AH (159.0 ± 6.1 cm) was similar to the TH (161.2 ± 4.6 cm) and to the AH predicted at the initial evaluation (160.8 ± 6.0 cm), and the actual AH correlated positively with both (R = 0.76, P = 0.0003; R = 0.71, P = 0.008, respectively).

The AH was below 152 cm in 7 girls, of whom 3 were characterized by paternal transmission of the advanced puberty. The AH was lower than the TH by >5.6 cm in 8 girls.

The AH (cm) could be calculated at the initial evaluation: 1.8822 age + 3.3510 height (SD) - 0.7465 bone age – 1.7993 pubic hair stage + 2.8409 TH (SD) + 150.32.

The formula is available online at http://www.kamick.org/lemaire/med/girls-advpub.html.

The calculated AH (159.0 ± 5.7 cm) and the actual AH were highly correlated (R = 0.93). The actual AH was lower than the calculated AH by > 0.5 SD in only one case (4.35 cm).

**Conclusion:**

We established a formula that can be used at an initial evaluation to predict the AH, and then to assess the risk of reduced AH as a result of advanced puberty. According to this formula, the actual AH was lower than the calculated AH by more than 2.8 cm (0.5 SD) in only one girl. The AHs of the untreated girls with advanced puberty did not differ from those predicted at the initial evaluation by the Bayley and Pinneau table or from the THs. However, this study provides a useful and ready-to-use formula that can be an additional assessment of girls with advanced puberty.

## Background

Advanced puberty in girls is defined as the onset of puberty between the ages of 8 and 10 yr. In nearly all cases, advanced puberty, which is a variant of normal puberty, is due to a familial condition rather than a pathological condition.

The premature secretion of estradiol increases the growth rate and accelerates bone maturation, which can shorten the growing period, and it may cause reduced adult height (AH). Treatment with gonadotropin-releasing hormone (GnRH) analog blocks the pituitary-ovarian axis, thereby preventing the secretion of estradiol, slowing the bone age (BA) progression and preserving growth potential. This action has been demonstrated in the evolving forms of idiopathic central precocious puberty in girls [1]. However, in two randomized studies on girls with onset of puberty at 7.5-8.5 yr [2] and at 8.4-10 yr [3], the AHs were similar between the untreated patients and the patients treated with GnRH analog. In a previous study on girls with advanced puberty [4], we demonstrated that the AHs of untreated patients and patients treated with GnRH analog were similar between the two groups. Furthermore, the participants’ AHs were similar to their target heights (TH) and were significantly below their heights at age 4 yr, as expressed by the standard deviation score (SDS). The AH was below the TH by more than 5 cm in 7/40 (17.5%) girls, 5 of whom were untreated and 2 of whom were treated. Determining the risk of reduced AH as a result of advanced puberty is of crucial importance because the age limit that is used to define central precocious puberty has decreased over time [5,6].

In the present paper, we studied 50 girls with idiopathic advanced puberty who reached their AH after undergoing spontaneous puberty. Data analysis was performed using multiple linear regression models. The objective was to predict the AH at the initial evaluation and to characterize the patients with an actual AH that was below -2 SD and/or lower than their TH by more than 1 SD.

## Methods

### Patients

This retrospective, single-center, cohort study was conducted on 50 girls who were monitored for idiopathic advanced puberty by a senior pediatric endocrinologist (R. Brauner) in a university pediatric hospital between 1996 and 2006 (all seen after our previous report) [4] and are currently over the age of 15 yr. The participants all reached their AH (growth during the preceding year of less than 1 cm in a menstruating girl) after spontaneous, untreated puberty. Advanced puberty was diagnosed according to the appearance of breast development between the ages of 8 yr and 10 yr, accompanied by the presence of pubic or axillary hair and/or a growth rate greater than 2 SDS during the year before a clinical evaluation (health record data).

The patients were identified by checking computerized hospital charts for the term “advanced puberty”. Among the 242 girls followed over the 10 years of the study, 90 girls were excluded for the following reasons: they had been examined only once (n = 16); they had been adopted (n = 6); they had a previously diagnosed intracranial abnormality (n = 4, hydrocephalus, Rathke cyst or hamartoma); they had an associated bone disease or intrauterine growth retardation or another disease responsible for short stature (n = 12); or they were treated with GnRH analog because of evolving puberty or advanced puberty that was not psychologically tolerated (n = 52). Until an acceptable predicted AH was reached, the patients were monitored every 6 months for clinical and BA evaluations if indicated, and they were subsequently followed by their physician each year until the AH was achieved. When the AH was not available in the hospital record, a letter was sent to the parents asking for the patient’s age and most recent height as indicated in their health records as well as the patient’s growth in the last year; these data were collected by the patients’ physicians. Some of these letters were returned to us because of a change of address (n = 29), and some parents did not respond (n = 73). At the initial evaluation, the characteristics of these 102 girls without the AH available and those of the 50 girls who were included in the study were similar except for the age at first menstruation, which was lower in the patients without a response (10.5 ± 0.54 yr) than in those included in the study (11.2 ± 1.6 yr, P < 0.005).

### Methods

The initial evaluation included the following data: age at the onset of breast development (reported), height chart, growth rate, weight, pubertal stage, BA, and the height of each parent (reported), as well as the age of the mother at her first menstruation. The following heights were collected and expressed as cm and SDS for chronological age: height at age 4 yr, height at the time of breast development, height at the initial evaluation, height at the first menstruation and AH. Magnetic resonance imaging was performed in 5 girls because of the absence of a family history of advanced puberty (mainly a mother who underwent menarche before the age of 11 yr), rapid pubertal development and/or symptoms such as headache. The imaging was normal in all of the subjects.

Height and body mass index (BMI, weight in kg/height in m squared) were expressed as SDS for chronological age [7,8]. For the AH, one SD corresponded to 5.6 cm [7]. The pubertal stage was calculated according to Marshall and Tanner [9]. The BA was assessed by R. Brauner according to the Greulich and Pyle method [10] in all of the participants. The TH was calculated based on the parental heights [11]. The predicted AH was calculated at the initial evaluation [12]; we used the column “advanced” when the BA advance was greater than one year.

To evaluate the difference induced by advanced puberty, the actual AH was compared to the TH, to the height at age 4 yr (SD) and to the AH predicted at the initial evaluation. Pubertal growth was calculated as the difference in cm between the height at the onset of breast development and the AH [13], and the duration of puberty was calculated as the time between this onset and the first menstruation.

Data analysis was performed using multiple linear regression models. An extensive analysis among the sets of variables (listed in Methods and Table 1) was conducted, and the models were validated based on the correlation of their predictions with the actual values. The robustness of the model was tested using cross-validation: the model was computed on a random, uniform sample of 80% of the patients and tested on the remaining 20%; this procedure was performed one hundred times on independently drawn samples.

**Table 1:** Characteristics of 50 girls with advanced puberty and spontaneous growth

	**n**	**Mean ± SD**	**Min**	**Max**
Age of onset of puberty, yr	50	8.9 ± 0.6	8	10
**Initial evaluation**				
Age, yr	50	9.8 ± 0.9	8.25	13.3
Bone age, yr	50	10.6 ± 1.4	8	16
Tanner stage of breast development	50	3.0 ± 1.0	1	5
Tanner stage of pubic hair development	50	2.7 ± 1.1	1	5
BMI, SD	49	0.8 ± 1.2	-1.78	3.92
**Evolution**				
Duration of puberty, yr	50	2.4 ± 0.9	0.7	4.0
Age at 1st menstruation, yr	50	11.2 ± 0.8	9.8	13.0
**Growth evolution**				
Height at onset of puberty, cm	48	132.7 ± 5.7	122.0	145.0
Height at initial evaluation, cm	50	140.30 ± 6.00	128.6	159.0
Height at 1st menstruation, cm	48	149.2 ± 5.6	137.0	162.0
Adult height, cm	50	159.0 ± 6.1	148.0	173.0
Target height, cm	50	161.2 ± 4.6	150.5	171.0
Predicted adult height, cm	50	160.8 ± 6.0	147.5	174.8
Adult – target heights, cm	50	-2.2 ± 4.0	-16.5	7.0
Adult height - height at 1st menstruation, cm	48	9.6 ± 3.6	2.5	17.0
Pubertal growth, cm	48	26.0 ± 4.7	15.0	39.0

## Results

### Characteristics of the population

The age at the onset of puberty was 8.9 ± 0.6 yr and 9.8 ± 0.9 yr at the initial evaluation, with the BA at 10.6 ± 1.4 yr (Table 1). The BMI was 0.8 ± 1.2 SDS. The duration of puberty was 2.4 ± 0.9 yr. The age at first menstruation in the girls was 11.2 ± 0.8 yr; this age was significantly younger than the corresponding age of their mothers at their first menstruation, which was 12.5 ± 1.6 yr (P < 0.00001). Nine mothers (18%) had undergone menarche before the age of 11 yr.

### Growth evolution and AH

**Figure 1:**
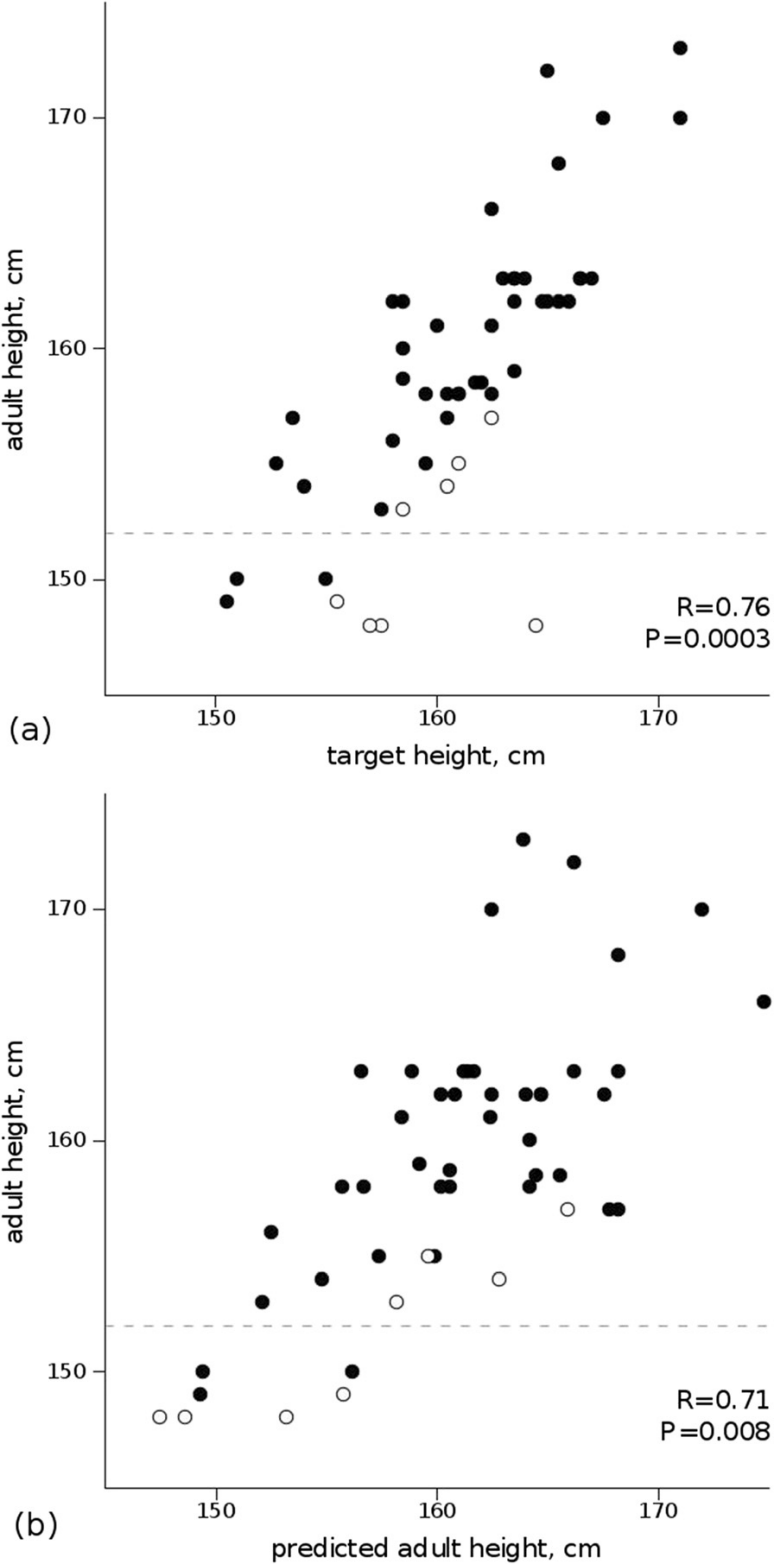
**Correlation between adult height and target height (a)/predicted adult height at the initial evaluation (b) in 50 girls with advanced puberty after spontaneous growth.** Open circles indicate those girls with an adult height lower than the target height by >5.6 cm (one SD). Dotted lines represent -2 SD (152 cm).

The AH was similar to the TH and to the AH predicted at the initial evaluation (Table 1 and Figure 1). The AH correlated significantly and positively with both the TH and the predicted AH.

Expressed as SDS, the AH was significantly lower than the height at age 4 yr (-0.77 ± 1.10 vs 0.52 ± 1.24 SDS), with a mean height loss of 1.32 SD. Pubertal growth correlated negatively with the age at the onset of puberty (R = -0.45, P < 0.01) and with the stages of breast development (R = -0.47, P < 0.01) and pubic hair development (R = -0.52, P < 0.001) at the initial evaluation.The AH was below -2 SD (152 cm) in 7 (14%) girls (Figure 1). For 3 girls, the father’s height was short (160 cm, 162 cm and 163 cm), and he had exhibited advanced puberty. Two girls were obese (BMI 2.22 and 2.53 SDS at the first evaluation). Pubertal growth was <20 cm in 4 of 7 girls. Diseases such as excessive androgen levels, hypothalamic-pituitary lesions (MRI normal, n = 3) and congenital bone disease (skeletal radiographies and genetic advice, n = 3) were excluded.

The AH was lower than the TH by more than one SD (5.6 cm) in 8 girls, including 4 of 7 girls with heights below -2 SD. Among the other 4 girls, one had severe scoliosis, and 3 were obese. Furthermore, among these girls, the AH SD was similar to the father’s height SD in 2 girls and similar to the mother’s height in 2 girls.

All 11 patients with an AH that was below -2 SD and/or lower than their TH by more than one SD had begun to menstruate after age 10.

### Prediction of the AH

The AH could be calculated at the initial evaluation, which was performed 1.0 ± 0.8 yr after the onset of puberty, using the following formula:

AH calculated (SD) = 0.3361 age + 0.5984 height (SD) - 0.1333 BA - 0.3213 pubic hair stage + 0.5073 TH (SD) – 2.3187 or using cm for AH as follows:

AH calculated (cm) = 1.8822 age + 3.3510 height (SD) - 0.7465 BA – 1.7993 pubic hair stage + 2.8409 TH (SD) + 150.32.

These formulae are available online at http://www.kamick.org/lemaire/med/girls-advpub.html.

**Figure 2:**
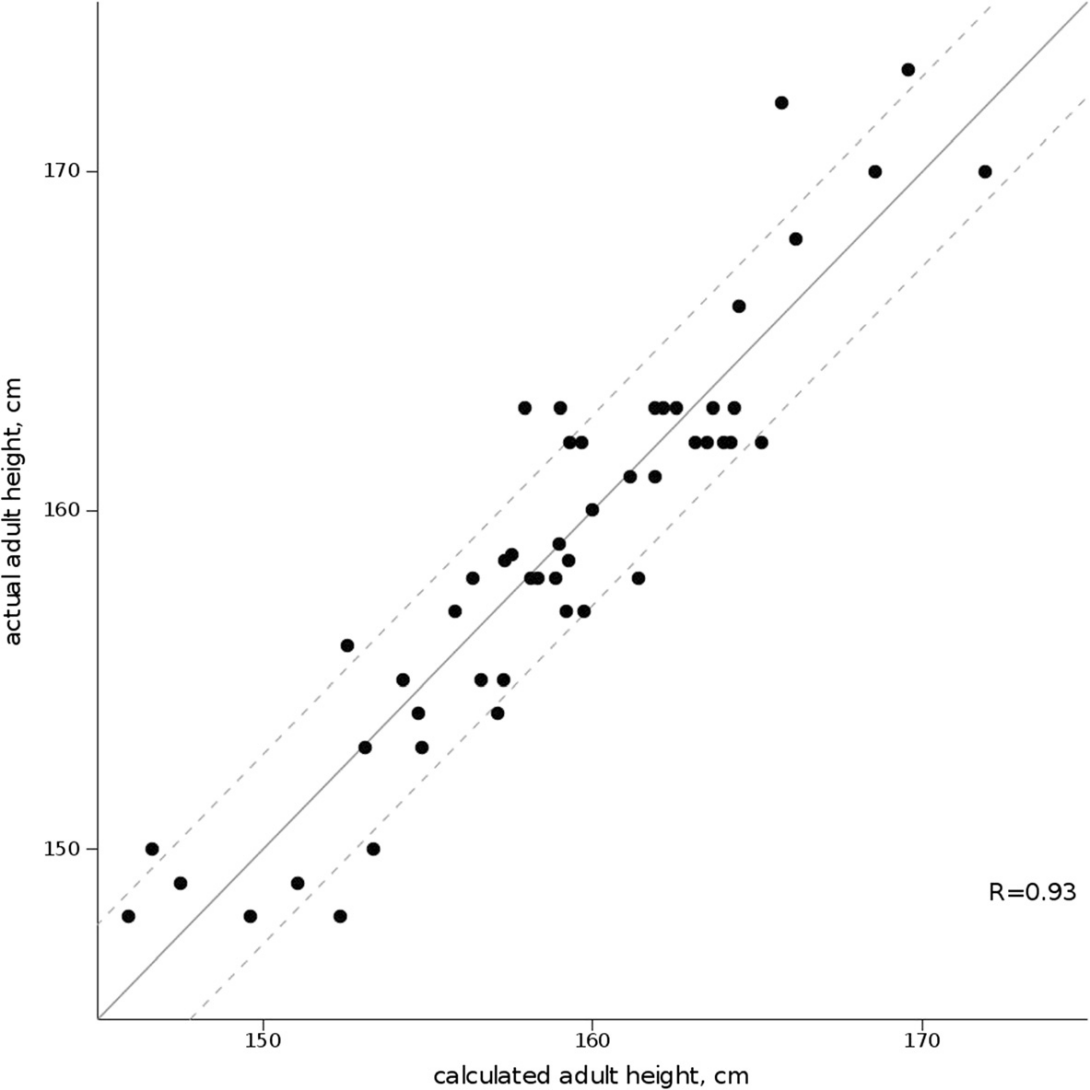
Correlation between actual adult height and calculated adult height in 50 girls with advanced puberty after spontaneous growth using the formulahttp://www.kamick.org/lemaire/med/girls-advpub.html. Plain line represents the reference perfect prediction (calculated = actual), and dotted lines represent ±0.5 SD from that value.

The data required are the age (yr) and height at the initial evaluation (cm or SD), the BA (yr), the Tanner stage of pubic hair development, and the heights of the father and mother (cm or SD).The calculated AH (159.0 ± 5.7 cm) and the actual AH were highly correlated (R = 0.93, Figure 2). The actual AH was different from the AH calculated by the formula by more than 5.6 cm (1 SD) in only one case, in whom the actual AH was greater, and by more than 2.8 cm (0.5 SD) in 11 cases (22%): the actual AH was greater than the calculated AH in 6 cases, just at the limit in 4 cases and lower by more than 2.8 cm in one case (4.35 cm). The quality of the above formula was confirmed by cross-validation (R = 0.90 on average).

## Discussion

We established a formula that can be used at an initial evaluation to predict the AH and then to assess the risk of reduced AH as a result of advanced puberty. According to this formula, the actual AH was lower than the calculated AH by more than 2.8 cm (0.5 SD) in only one girl, with a difference of 4.35 cm.

### Formula for prediction

The formula that can be used at the initial evaluation to predict the AH includes the TH and data obtained during this evaluation (chronological and bone ages, height (SD) and pubic hair development stage). In two previous studies, we used mathematical models to improve the diagnosis of growth hormone deficiency (GHD) [14] or to predict the AH in girls with idiopathic central precocious puberty [15]. Using logical analysis data [14], we have shown that the screening of GHD can be achieved by employing a simple graph based on insulin-like growth factor (IGF) 1 and the growth rate: (1) all patients below a given line (growth rate ≤ -7.3 – 1.3 × IGF (SDS)) had GHD and pituitary stalk interruption syndrome; (2) all but two patients above another given line (growth rate > -4.5 + 6.4/(IGF + 4.5) (SDS)) did not have GHD; and (3) in-between, patients in a “gray area” could not be diagnosed using only the growth rate and IGF I. The only two out of 54 patients with GHD who had been misdiagnosed had an abnormal BMI. Using multiple linear regression [15], we showed that in girls with untreated idiopathic central precocious puberty, the difference between the AH and TH (1.7 ± 4.3 cm) can be predicted at an initial evaluation as follows: 2.76 (height at initial evaluation - TH) - 3.68 LH/FSH peaks ratio - 3.49; R = 0.88. These formulae are available at http://www.kamick.org/lemaire/med/girls-cpp.html. The actual AH of 9 girls (17%) was >3 cm lower than the AH predicted by the formula. Four (44%) of the 9 girls had a BMI >2 SDS, whereas 6 girls (66%) had a BA advance greater than 2 years at the initial evaluation; the corresponding percentages were 30.8% and 26.9% for the entire group. In the present study on older girls with advanced, and not precocious, untreated puberty, only one girl had an actual AH that was more than 3 cm (4.35 cm) lower than the calculated AH. The formula includes the TH and chronological and bone ages, height (SD) and pubic hair stage. The GnRH test result is not included in the formula because this test has not been performed in girls with advanced puberty. The lower number of patients with a significant difference between the calculated and actual AHs is likely due to the proximity to the end of growth. In our three studies on patients who were followed by R. Brauner (GHD, central precocious puberty and advanced puberty), most misclassifications were associated with an abnormal BMI. These misclassifications likely occurred because increased BMI is associated with earlier pubertal development [16,17], namely, earlier pubic hair development [18], an increased growth rate and BA progression [19].

### Does advanced puberty decrease the growth potential?

In the present study, the AH was not different from the TH or from the AH predicted at the initial evaluation, and the AH correlated significantly and positively with both the TH and the predicted AH. This observation has been reported in previous studies on untreated girls with central precocious puberty [20] and advanced puberty [2,3,21]. Additionally, these studies have shown that the AH is not different between untreated girls and girls treated with GnRH analog.

The AH was below -2 SD (152 cm) in 14% of the girls. What are the factors that contributed to this short height, and can we predict them? What are the roles of obesity and the increase of insulin in the progression of bone maturation? What is the role of the transmission of advanced puberty by the paternal family, and not the maternal family, as typically observed in precocious puberty [22]?

Bar et al. [20] reported that only 10% of untreated girls with idiopathic precocious puberty had an AH < 150 cm (1st percentile, -2.3 SD), whereas 90% of untreated girls achieved a normal height, which is slightly less than the average for healthy American girls (163.8 cm). These authors also observed that 75% of the group achieved a height within 6.3 cm of the initial predicted AH. Lazar et al. [21] reported that 67% of untreated girls with advanced puberty achieved or surpassed the TH range (TH ± 0.5 SD). In the present study, the corresponding percentage was 52%.

We aimed to compare the AH to the height at age 4 yr (SD) for two reasons. First, the differences in height that are due to genetic factors occur before this age, between birth and approximately 2.5 to 3 yr of age. Second, the height at the initial evaluation, expressed as the SD, is influenced by sex steroid secretion, which does not affect the growth rate at age 4 yr. We therefore found that the height (SD) at the initial evaluation was significantly greater than that at age 4 yr.

### What are the mechanisms that compensate for the decrease in growth potential?

In the present study, the increases in the duration of puberty and pubertal growth were sufficient to compensate for the decrease in height induced by the premature secretion of estradiol, which accelerates the progression of BA.

Thus, the duration of puberty was greater than the normal duration (2 yr) in the present study (2.4 yr) and in previously reported studies (4.9 yr [20], 2.45 yr [21]), 3.2 yr [23]). In our earlier study on the prediction of AH in girls with central precocious puberty [15], the duration of puberty could be predicted using a formula available at http://www.kamick.org/lemaire/med/girls-cpp.html. The duration of puberty was longer in girls who were youngest at the onset of puberty and in those who had a smaller difference between the height at the initial evaluation and the TH. Marti-Henneberg et al. [24] showed a negative correlation between the age at the onset of puberty and the duration of puberty and between the age at the onset of puberty and the age at first menstruation. Rosenfield et al. [25] reported slightly early menarche (0.5 yr) despite thelarche that occurred 1.3 yr early.

The height gain between the onset of puberty and the achievement of the AH (26.0 ± 4.7 cm) was greater than that reported by Tanner et al. [13] during normal puberty (25 cm). In the present study, the girls had gained more height between the first menstruation and the achievement of the AH (9.6 cm) than girls who had first menstruated at the age of 13 yr (7 cm) [26].

### Study limitations

This study has several limitations. It is retrospective and limited to 50 girls. The girls who were excluded because of the change in their address may introduce bias. We postulate that the similarity of these girls to the girls who were included with regard to the variables analyzed limits this bias. The AHs of only a few of the girls were collected from health records held by their pediatricians, and the height reported by parents is less accurate than the measured height. The major limitation is the lack of validation of the formula on a separate patient population. However, this study provides a useful and ready-to-use formula for AH prediction.

## Conclusion

Advanced puberty is a variant of normal puberty and does not typically require medical care. Its occurrence in children with short stature may accentuate the deficit. We established a formula that can be used at an initial evaluation to predict the AH and then to assess the risk of reduced AH as a result of advanced puberty. The AHs of the untreated girls with advanced puberty did not differ from those predicted at the initial evaluation by the Bayley and Pinneau table or from the THs. However, this study provides a useful and ready-to-use formula that can be used as an additional assessment for girls with advanced puberty. We advise that additional investigations be conducted to validate our predictions in studies at other institutions.

### Statement of the ethical review committee

The Ethical Review Committee (Comité de Protection des Personnes, Ile de France III) approved this retrospective study and stated that “This study appears to be in accordance with the scientific principles generally accepted and to the ethical standards of research. The study was lead in the respect of the French law and regulation”.

## Abbreviations

AH: Adult height; BA: Bone age; BMI: Body mass index; GHD: Growth hormone deficiency; GnRH: Gonadotropin-releasing hormone; IGF: Insulin-like growth factor; SDS: Standard deviation score; TH: Target height.

## Competing interests

The authors declare that they have no competing interests.

## Authors' contributions

PL analyzed the data, prepared the Table and Figures and participated in the preparation of the manuscript. DP and J-BB participated in the conception and design and the acquisition of data. RB directed the work and prepared the manuscript. All authors read and approved the final manuscript.

## Pre-publication history

The pre-publication history for this paper can be accessed here:

http://www.biomedcentral.com/1471-2431/14/172/prepub
